# How Do Children Solve Aesop's Fable?

**DOI:** 10.1371/journal.pone.0040574

**Published:** 2012-07-25

**Authors:** Lucy G. Cheke, Elsa Loissel, Nicola S. Clayton

**Affiliations:** Department of Experimental Psychology, University of Cambridge, Cambridge, United Kingdom; University of Queensland, Australia

## Abstract

Studies on members of the crow family using the “Aesop's Fable” paradigm have revealed remarkable abilities in these birds, and suggested a mechanism by which associative learning and folk physics may interact when learning new problems. In the present study, children between 4 and 10 years of age were tested on the same tasks as the birds. Overall the performance of the children between 5–7-years was similar to that of the birds, while children from 8-years were able to succeed in all tasks from the first trial. However the pattern of performance across tasks suggested that different learning mechanisms might be being employed by children than by adult birds. Specifically, it is possible that in children, unlike corvids, performance is not affected by counter-intuitive mechanism cues.

## Introduction

Recent studies with members of the crow family [1], [2], [3] have investigated the cognition underlying one of Aesop's most familiar fables. In this tale, a thirsty crow comes across a half-filled jug of water. Unable to reach the water to drink, the crow drops stones into the pitcher until the level of the water raises enough for him to drink. Bird and Emery [1] found that Rooks were not only capable of this, but would choose the most efficient tool (large rather than small stones) and preferentially drop stones into water rather than sawdust. Subsequent studies with Eurasian Jays [2] sought to investigate whether another species of corvid could also solve this task and if so, what these birds understand.

After learning to drop stones into a tube to receive a reward, the Jays were presented with a choice between a tube half-filled with sawdust and a tube half-filled with water, both containing an out-of-reach food item. Having had no experience of water in this form, or discovering the consequences of dropping stones into water, this choice could be used to assess the birds' ability to learn the necessary conditions for success. Two out of the four birds that were tested learned to drop significantly more stones into the water than into the sawdust over the course of 15 trials (the others were uninterested in the task). These two birds also quickly learned to drop significantly more sinking than x floating items into water. Such learning, while impressive, cannot be said to differ in nature to that characteristic of instrumental learning, namely that performance of a particular action (in this case dropping a sinkable item into water) leads to the increased probability of a reward. For example, in the sawdust versus water task, dropping a stone into water would lead to the food reward being reachable on around a fifth of occasions, while dropping a stone into sawdust would never result in the reward being accessible. Thus the birds may simply have learned which tube was more likely to be rewarded. Dropping a stone into water would also lead to the food moving slightly closer, which may also be rewarding, while dropping a stone into sawdust leads to no movement of the reward.

To investigate whether the birds' performance could be explained by instrumental learning, Cheke and colleagues conducted a series of control tests that showed that the birds were able to learn in a mechanised version of the task in which stone-dropping resulted in the approach of food. The jays were, however, unable to learn when the reward probabilities remained the same, but the reward did not *move*. This contrast was interpreted to suggest that it was not the causal mechanism of the Aesop's Fable task that the birds were able to learn, but the relationship between stone-insertions and movement. In a final control, Cheke and colleagues presented the birds with a “U-tube” apparatus in which insertion of a stone into the correct tube apparently caused the level of water in the adjacent tube to rise. This apparatus consisted of a U-tube and a single tube, whose bases were hidden beneath an opaque base. Because the U-tube contained a single body of water, a stone inserted into one arm would raise the level of water in both arms, while a stone inserted into the single tube would raise the level of only that tube. The bait was placed within one arm of the U-tube. This task was designed to offer the same movement cues as the original task (i.e. stone insertion into one tube caused the approach of food, stone insertion into the other tube did not) but with confusing or counterintuitive mechanism cues. Cheke and colleagues found that the birds were unable to learn this task even when given twice as many trials as on the original task. They thus suggested that while instrumental learning involving movement cues was both necessary and sufficient for learning in the Aesop's fable task, the presence of cues suggesting a “possible” or “impossible” causal mechanism were able to enhance or retard learning respectively.

The Aesop's Fable paradigm provides a valuable tool with which to investigate the interaction between instrumental learning (the ability to learn to perform an action if that action is rewarded), causal reasoning (understanding *that* one event causes another) and mechanistic inference (understanding *why* one event causes another, i.e. the ability to explain the causal relationship between two things in terms of the underlying mechanism). An advantage of this particular paradigm is that the mechanism is natural (i.e. not man-made) and perceivable (in that it doesn't involve invisible forces such as electricity or magnetism), and the action (stone dropping) is physically simple. This means that the ability to understand the affordances of the task can be easily separated from exposure to technology and fine motor capacity and thus makes it appropriate for investigating physical cognition in both young children and animals.

Much research on causal reasoning in children has employed paradigms in which the to-be-inferred mechanism is explicitly explained and primed [4] or is opaque [5], [6], [7], [8], [9]. As such it does not allow investigation into the interaction between causal and mechanistic reasoning. Such studies reveal that children of 3–4 years are able to infer causation from co-variation and contiguity (and many other factors such as temporal order and reasoning by exclusion). Even infants are capable of forming expectations from complex statistical regularities [10], [11], [12], although they are unable to act upon them. However, these studies cannot assess the extent to which children are making inferences about the mechanism. Our aim is to investigate the interaction between causal understanding based on instrumental learning (that is, based on contingency and contiguity) and understanding of mechanism in the learning of physical problems. Further, we wish to explore whether cognitive systems as structurally divergent as those of corvids and humans can be said to be functionally convergent in terms of not only their *performance* on physical cognition tasks, but also in the manner in which these tasks are learned.

It has been argued that causal reasoning based on statistical and perception-based analysis may develop separately to mechanism-based analysis. These two systems interact to allow children to flexibly adapt their causal models of the world by neither being led too often down blind alleys by coincidental contingency, or being prevented from identifying causation in situations involving unfamiliar mechanisms [13]. Thus one might expect children to pass through several stages of understanding as they develop; from being unable to learn about the relationship between actions and consequences, through having a concept of causality based on perceptual and statistical regularities only, to having a concept of mechanisms that can be adapted and finessed by perceived causal relationships (for example, understanding that unsupported things *usually* fall but being able to learn that this is not true if those things have wings).

In the current experiment, children between the ages of 4- and 10-years were trained to drop stones into tubes in a similar manner as the Eurasian Jays [2]. They were presented with three of the same tasks as the birds: Water versus Sawdust [1], [2], [3], Sinking versus Floating [2], [3], and the U-tube [2]. In all tasks the children were given five 2-minute trials in which to attempt to retrieve a floating token that could be exchanged for a sticker. Finally, these results were compared to performance on the classic Piagetian conservation of volume task, which has been classically used to differentiate between pre-operational and concrete operational thought in children. The former is associated with “phenomenistic” reasoning (inferring causation from co-occurrence) while the latter is associated with reasoning about seen or inferred mechanisms. This transition is thought to occur around the age of 7-years [14], [15], [16].

We hypothesised that the children would pass through several stages of performance during development (see Table 1). Specifically, we predicted that many 4-year-olds would be unable to learn any of the tasks and would perform at chance (due to the complex cause-effect relationships involved), whereas the slightly older children (older 4-year-olds, 5-year-olds and 6-year-olds) would be able to learn the association between dropping particular items in particular locations and an approaching reward. This is based on literature suggesting that children of this age can learn and act on cause-effect relationships [5], [6], [7], [8], [9], [12]. These children will not, however, have a concept of the causal mechanism and consequently they would perform equally well on all three tasks. We predicted that older children (7-/8-/9-year-olds) would have formed a (potentially simplified) concept of the mechanism underlying displacement (as predicted by previous studies on causal mechanism [13], [16], [17], [18]). These children should have a pre-formed idea about what is and is not possible given this mechanism. Consequently, these children should perform comparably on the first two tasks, but perform badly on the U-tube since this task presents an apparently impossible causal relationship. Finally, the oldest children (10-years) may be able to flexibly adapt their inferred mechanism and potentially infer the presence of the U-tube so as to allow them to marry the perceptual contingency with their understanding of mechanism. These children were thus expected to perform with a very high success rate on all three tasks. Finally, we conjectured that performance on tasks 1 and 2 should be predicted by performance on the conservation of volume task [14], [15], [16].

**Table 1:** Predicted performance of children of different age groups.

	Age	Learning Ability	Type of rule that can be learned	Predicted performance		
		(as predicted by literature)		Task 1 Water/ Sawdust	Task 2 Sinking/ Floating	Task 3 U-tube
Able to adjust expectations from statistical regularities, but unable to act on this information	8 months –4 years	Babies as young as 8 months are surprised when statistical regularities are violated, but are not capable of acting on this information.[10], [11], [12]	Normally, when I see X, then I see Y.	✗	✗	✗
Able to use covariation to infer causal relationships	4–6	4 Year-Olds able to infer causal relationships using Covariation and Contiguity and act upon them. [5], [6], [7], [8], [9], [12] (but examples used were easier cause-effect relations that those presented here)	If I do X, then Y happens.	✓	✓	✓
Able to infer underlying mechanism	7–9	7–9 Year-Olds able to come up with intuitive novels solutions and reason in terms of mechanisms. [14], [16], [17], [18]	If I do X, then Y happens, because…	✓	✓	✗
Able to flexibly understand underlying mechanism	10		Normally if I do X, then Y happens, but not when Z because	✓	✓	✓

## Methods

### Subjects

Children aged 4–10 (N = 80: 4-year-olds: n = 20; 5-year-olds: n = 16; 6-year-olds: n = 4; 7-year-olds: n = 14; 8-year-olds: n = 11; 9-year-olds: n = 8; 10-year-olds: n = 5) were recruited from a Cambridgeshire Primary School. The sample consisted of 40 boys and 42 girls. Two subjects (both boys: one 4-year-old and one 5-year-old) were removed from the analysis because they were unwilling to take part in the experiment.

### Ethics Statement

This study was approved by the University of Cambridge Research Ethics Committee. Informed written consent was gained from parents before any child took part.

### Procedure

Subjects were tested individually in a room in the school. Each subject was presented with a series of tasks that were equivalent to those used by Cheke and colleague's recent experiments with Eurasian Jays [2].

### Training

**Figure 1:**
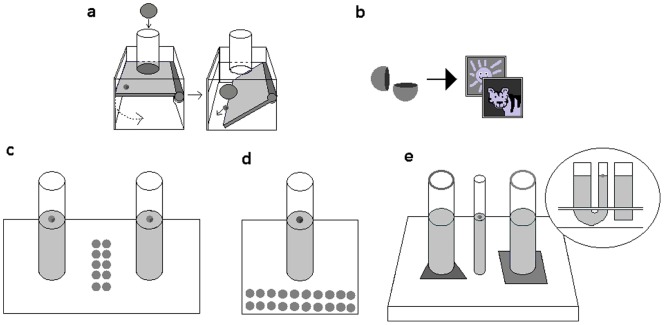
Schematic of the apparatuses used. 1a. Training Apparatus. When a stone is dropped into the tube, the platform drops and the token is released. 1b. Tokens. When a token is retrieved, it can be swapped for a sticker of the child's choice. 1c. Task 1: Water versus Sawdust. When a marble is dropped into the water, the level of the floating token rises. When a stone is dropped into the sawdust, the level of the token does not change. 1d. Task 2: Sinking versus Floating. When a marble is dropped into the water, it sinks and the level of the floating token rises. When a cork ball is dropped into the water it floats and the level of the token does not change. 1e. Task 3: U-tube. When a stone is dropped into the U-tube, the level of the floating token rises. When a stone is dropped into the separate tube, the level of the token does not change.

The children were presented with the “platform” apparatus originally used by Bird and Emery [19] and subsequently by Cheke and colleagues [2] consisting of a Perspex box in which a platform is held in place by a magnet. When a heavy object is dropped down a tube in the top of the box, the platform is released and anything resting on it is released from the box [see fig 1]. The children were shown a small red token and informed that these could be exchanged for stickers. The token was then placed onto the platform and the children were shown a bowl of blue stones. If the children did not spontaneously drop the stone down the tube, they were encouraged to do so by the experimenter demonstrating this action to them. Training was completed when the children had dropped a stone into the apparatus, retrieved the token and swapped it for a sticker twice.

The following experiments were conducted as near as possible to the way that the corvids were tested so as to maintain the possibility for direct comparisons between the studies. For this reason, tasks were presented in a specific order which was not counterbalanced between subjects. In all tasks, the children were informed that if they could retrieve the/one of the tokens they could swap it for a sticker. To prevent them from reaching their entire arm into the tube/tubes, the children were also told that they were not allowed to put their thumb in the tube. To ensure that the objects used were “novel” and that previous knowledge about the specific properties could not affect performance and reduce the need for task-specific learning, all objects used were painted to disguise their material. The children were given 5 trials. If they did not spontaneously insert any items they were prompted with “You are allowed to try anything you like” or “why not just try things” or “try to get the/one of the tokens”. Approximately 7 items were needed to retrieve the token, although this varied depending on children's token-retrieval technique. Trials were ended after two minutes or if the children inserted all available items. If children had an error-free performance, including successfully retrieving the token, on three consecutive trials they were deemed to fully understand the task and were not tested further (so as to prevent them from losing interest). Number and location of item insertions were recorded, as well as success in retrieving the token. Number of item insertions was used as the dependant variable, token retrieval is reported in Supporting Information S1.

### Task 1: Water versus Sawdust

The children were presented with two Perspex tubes (inner diameter 5 cm, outer diameter 6 cm, height 18 cm), one that contained water and the other containing sawdust. A token was placed into both tubes such that it rested on the surface of the water/sawdust and was approximately 2 cm from the children's reach. Between the tubes was placed a bowl containing ten red marbles of 2 cm diameter. Between trials, the positions of the two tubes were exchanged pseudo-randomly.

### Task 2: Sinking versus Floating

The procedure was identical to Task 1 except that a single water-filled tube (inner diameter 5cm, outer diameter 6cm, height 18 cm) was presented beside a bowl containing 10 yellow cork balls (1.5g) and 10 yellow marbles (15g). These were visually indistinguishable in size (2cm diameter) and colour (yellow), but differed in density and weight.

### Task 3: U-tube

The children were presented with an apparatus consisting of one U-shaped tube with one wide arm (3 cm inner diameter) and one narrow arm (1.3 cm inner diameter), and a single wide tube (3 cm inner diameter). These were embedded in an opaque base such that the join of the U-tube was hidden and the apparatus appeared to consist of two identical wide tubes with a narrow tube between them (as shown in Fig. 1). Both tubes were filled with water such that the level was equal between them and at least 1cm from the aperture of the narrow arm of the U-tube. The base of each wide tube was marked with a different coloured shape.

The children were presented with ten blue stones. These could be inserted into the wide arm of the U-tube or the single wide tube, but were too large to fit into the narrow arm of the U-tube. A token was placed into the narrow tube (i.e. the narrow arm of the U-tube). After the final trial, the children were asked “how do you think this works?” Children's answers were coded into four categories: “No Explanation” consisted of silence, “I don't know” or irrelevant responses, “Descriptive Explanation” consisted of an accurate description of the relationship between the children's actions and the movement of the token, “Inference Explanation” consisted of an inference about a hidden connection between two of the tubes, and “Mechanistic Explanation” consisted of answers mentioning that the water was *displaced* by the dropping of the stone.

### Piagetian Conservation Task

To relate the results of this experiment to classic tasks, children were also tested on the Piagetian conservation of volume task (e.g. [15]). Children were presented with a tall thin container and a short wide container and witnessed water being poured from one to the other. They were then asked whether the amount of water was now the same, more or less than it had been. The order in which these options were presented was counterbalanced between children. This methodology was chosen over more common methods to prevent possible confounds resulting from repeated questions [20].

### Analysis

Because the majority of the data to be analysed is in the form of proportions (number of correct actions out of total actions) statistical analysis was mostly nonparametric. Performance was compared to chance using one-sample Wilcoxon signed rank tests. The average age of different groups was compared using independent samples t-tests. Performance was correlated with other metrics using Kendall's Tau. Performance across tasks was modelled using Generalized Estimating Equations with a binomial logistic response type and generalized Chi statistic. Post-hoc tests were conducted using Mann-Whitney U and Wilcoxon tests with Šidák alpha correction for multiple comparisons.

## Results

### Task 1: Water versus Sawdust

All 80 children took part in this task. Eight children completed 3 consecutive error-free trials (in which they only inserted stones into the tube containing water). Four of these children (one 7-year-old, two 8-year-olds and one one 10-year-old: mean age 8.48) showed mistake-free performance from the first trial. A further four children (one 5-year-old, one 7-year-old, one 8-year-old and one 10-year old: mean age 8.41) showed perfect performance from the second trial onwards. While these children were not tested for the subsequent trials, their performance was extrapolated for the purposes of analysis.

**Figure 2:**
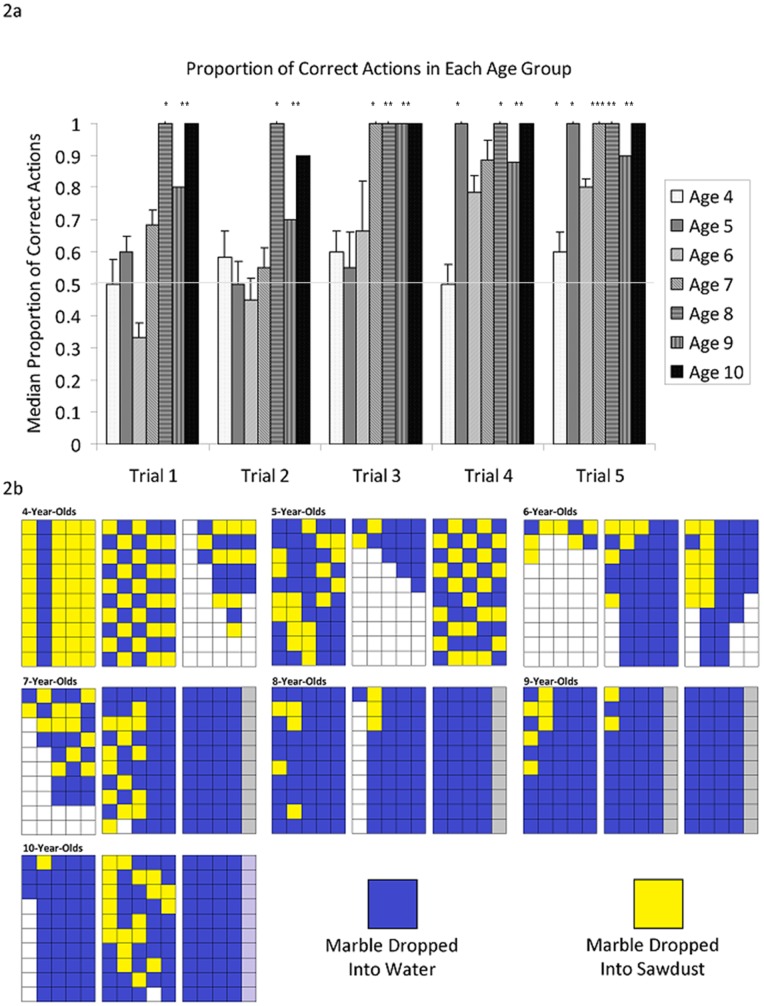
Performance of Children on Task 1. 2a. shows the median proportion of children of different age groups across the five trials of Task 1. Error bars represent 95% confidence intervals. 2b shows the individual marble insertions of 3 children chosen at random from each age cohort. Each column represents the order in which items were inserted within a single trial. Grey columns indicate trials not performed due to error-free performance in the three previous trials. Stars represent trials in which that age group performed above chance according to one-sample wilcoxen (4-year-olds: (1^st^: W = 1, n = 9, p>0.05; 2^nd^: W = 36, n = 12, p>0.1; 3^rd^: W = 38, n = 12, p>0.1; 4^th^: W = 29, n = 10, p>0.1; 5^th^: W = 71, n = 13, p<0.05; 5-year-olds: 1^st^: W = 12, n = 8, p = >0.05, 2^nd^: W = 7, n = 10, p>0.5, 3^rd^: W = 29, n = 12, p>0.1, 4^th^: W = 63, n = 14, p = 0.05; 5^th^: W = 82, n = 15, p<0.05; 7-year-olds: 1^st^: W = 28, n = 9, p>0.05; 2^nd^: W = 11, n = 9, p>0.05, 3^rd^: W = 41, n = 9, p<0.02; 4^th^: W = 59, n = 14, p>0.05; 5^th^: W = 100, n = 14, p<0.001; 8-year-olds: 1st: W = 52, n = 11, p<0.05; 2nd: W = 45, n = 11, p<0.05; 3rd: W = 66, n = 11, p<0.005; 4th: W = 52, n = 11, p<0.05; 5th: W = 66, n = 11, p<0.005; 9/10-year-olds: 1st: W = 75, n = 12, p<0.005; 2nd: W = 45, n = 11, p<0.002; 3rd: W = 78, n = 12, p<0.005; 4^th^: W = 82, n = 13, p<0.005; 5th: W = 78, n = 12, p<0.005).

Overall, performance improved gradually with age and reached a plateau at 8years (Fig. 2a). Children aged 4–7 years gradually learned over 5 trials which was the correct tube in which to insert marbles. The proportion of stones inserted into the correct tube was compared to a chance level of 0.5 for each age group for each trial using one-sample Wilcoxon signed rank tests (see figure 2 for statistics). Due to sample size constraints, performance against chance was not calculated for the 6-year-olds and the 9/10-year-old groups were combined. 4-year-olds performed better than chance on their 5th trial only. 5-year-olds performed better than chance on their 4th and 5th trials. 7-year-olds performed better than chance on their 3^rd^ and 5th trials. By contrast, 8- and 9/10-year-olds all performed better than chance on the 1st trial and all subsequent trials.

Age correlated positively with the proportion of marbles inserted into the correct tube on all trials (Kendall's tau: trial 1: R(61) = 0.302, p<0.005; trial 2: R(71) = 0.204, p<0.05; trial 3: R(77) = 0.260, p<0.005; trial 4: R(78) = 0.182, p<0.05; trial 5: R(76) = 0.244, p<0.01). There was a significant effect of age-group on proportion of marbles dropped into water in the first trial (Kruskal Wallis test: p<0.05) but not in any subsequent trials. Post-hoc pair-wise comparisons (Mann-Whitney U test with Šidák correction) indicated no significant differences between any specific age-groups. Taken together, these results suggest that by the age of 8 years, children know that dropping a marble into water will cause the level to rise while those aged between 4- and 7-years are able to learn this over the course of 5 trials.

### Task 2: Sinking versus Floating

All 80 children took part in this task. Thirteen children (one 5-year-old, two 7-year-olds, seven 8-year-olds and three 9-year-olds, mean age 8.23) solved the task without mistakes (i.e. inserted only sinkable items into the tube) on 3 consecutive trials, although none of them did so from the first trial. While these children were not tested for the subsequent trials, their performance was extrapolated for the purposes of analysis.

**Figure 3:**
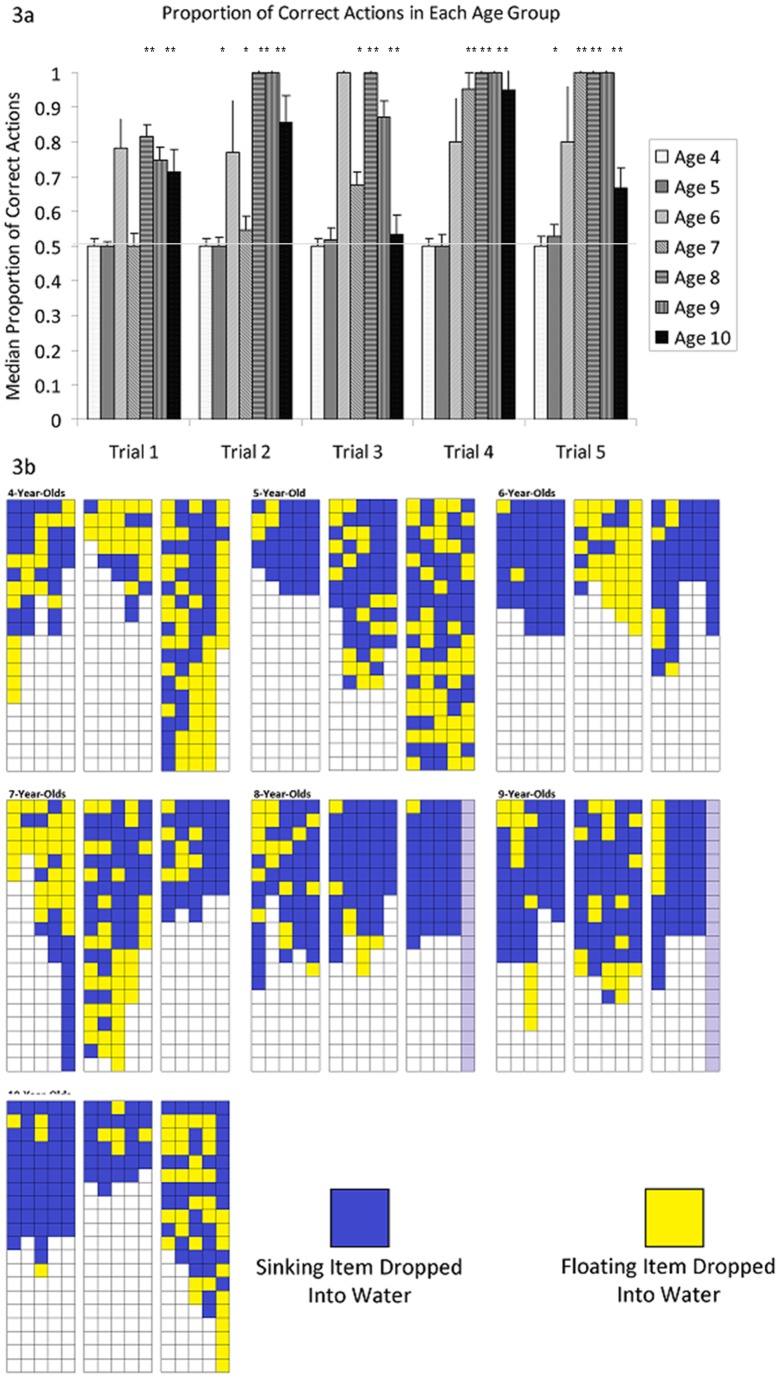
Performance of Children on Task 2. 3a. shows the median performance of children of different age groups across the five trials of Task 2. Error bars represent 95% confidence intervals. 3b shows the individual items inserted by 3 children chosen at random from each age cohort. Each column represents the order in which items were inserted within a single trial. Grey columns indicate trials not performed due to error-free performance in the three previous trials. Stars represent trials in which that age group performed above chance according to one-sample wilcoxen (4-year-olds: 1^st^: W = −15, n = 9, p>0.05; 2^nd^: W = −3, n = 6, p>0.05; 3^rd^: W = 7, n = 8, p>0.05; 4^th^: W = 29, n = 9, p>0.05; 5^th^: W = 14, n = 9, p>0.05; 5-year-olds: 1^st^: W = −8, n = 5, p>0.05; 2nd: W = 32, n = 8, p = 0.05; 3^rd^: W = 37, n = 10, p>0.05; 4^th^: W = 6, n = 8, p>0.05; 5th: W = 35, n = 9, p = 0.05; 7-year-olds: 1^st^: W = 17, n = 8, p>0.05; 2^nd^: W = 36, n = 9, p = 0.05; 3^rd^: W = 49, n = 10, p<0.02; 4^th^: W = 62, n = 11, p<0.01; 5^th^: W = 64, n = 11, p<0.005; 8-year-olds: 1^st^: W = 55, n = 10, p<0.01; 2^nd^: W = 53, n = 10, p<0.01; 3^rd^: W = 45, n = 9, p<0.005; 4^th^: W = 55, n = 10, p<0.01; 5^th^: W = 55, n = 10, p<0.01. 9-/10-year-olds: 1^st^: W = 45, n = 9, p<0.005; 2^nd^: W = 66, n = 11, p<0.005; 3^rd^: W = 55, n = 10, p<0.01; 4^th^: W = 66, n = 11, p<0.005; 5^th^: W = 60, n = 12, p<0.02).

As in the first task, performance improved gradually with age, although it seemed to drop in the oldest children (Fig. 3a). The proportion of stones inserted into the correct tube was compared to a chance level of 0.5 for each age group for each trial using one-sample Wilcoxon signed rank tests (exact statistics reported in figure 3). Due to sample size constraints, performance against chance was not calculated for the 6-year-olds and the 9/10 year old groups were combined. 4-year-olds did not perform above chance in any trial, 5-year-olds performed above chance in the 2nd and 5th trials, 7-year-olds performed above chance in the 2nd trial and all subsequent trials and 8- and 9-/10-year-olds performed above chance in all trials.

Age correlated significantly with performance in all trials (Kendall's Tau: 1^st^: R(78) = 0.422, p<0.001; 2^nd^: R(77) = 0.458, p<0.001; 3^rd^: R(78) = 0.344, p<0.001; 4^th^: R(77) = 0.416, p<0.001; 5^th^: R(78) = 0.372, p<0.001). There was a significant effect of age in all trials (Kruskal-Wallis tests: trial 1: p<0.001; trial 2: p<0.001; trial 3: p<0.001; trial 4: p<0.001; trial 5: p<0.001). Post-hoc pairwise comparisons revealed a significant difference between 4- and 8-year-olds on all trials (Mann-Whitney U test with Šidák correction, trial 1: p<0.001; trial 2: p<0.001; trial 3: p<0.001; trial 4: p<0.001; trial 5: p<0.001) between 4- and 9-year-olds on all trials (Mann-Whitney U test, Šidák correction, trial 1: p<0.003; trial 2: p<0.001; trial 3: p<0.001; trial 4: p<0.001; trial 5: p<0.003), between 5-and 8-year-olds on trials 1, 3, 4 and 5 (Mann-Whitney U test with Šidák correction, trial 1: p<0.001; trial 3: p<0.001; trial 4: p<0.001; trial 5: p<0.003) and between 5- and 9-year-olds on trials 2 and 4 (Mann-Whitney U test with Šidák correction, trial 2: p<0.001; trial 4: p<0.001). Taken together these results suggest a similar developmental trajectory to that found for the first task, namely that by the age of 8 years, children know that dropping a sinking, rather than a floating, object into water will cause its level to rise. In common with the first task, we also found that younger children, specifically those aged between 5- and 7-years, are able to learn this over the course of 5 trials. Unlike the first task, however, the 4-year-old children appear unable to learn the sinking versus floating task within 5 trials.

### Task 3: U-tube

Some children took part in another experiment (not reported) instead of the U-tube task, which is why sample size was reduced to Sixty-four children for this task. Of these, four children solved the task without mistakes (inserted no stones into the single tube) on 3 consecutive trials. One child (age 9.37) performed mistake-free from the first trial. Three children (one 5-year-old, one 8-year-old and one 10-year-old: mean age 8.2) performed mistake-free from the second trial. While these children were not tested for the subsequent trials, their performance was extrapolated for the purposes of analysis.

**Figure 4:**
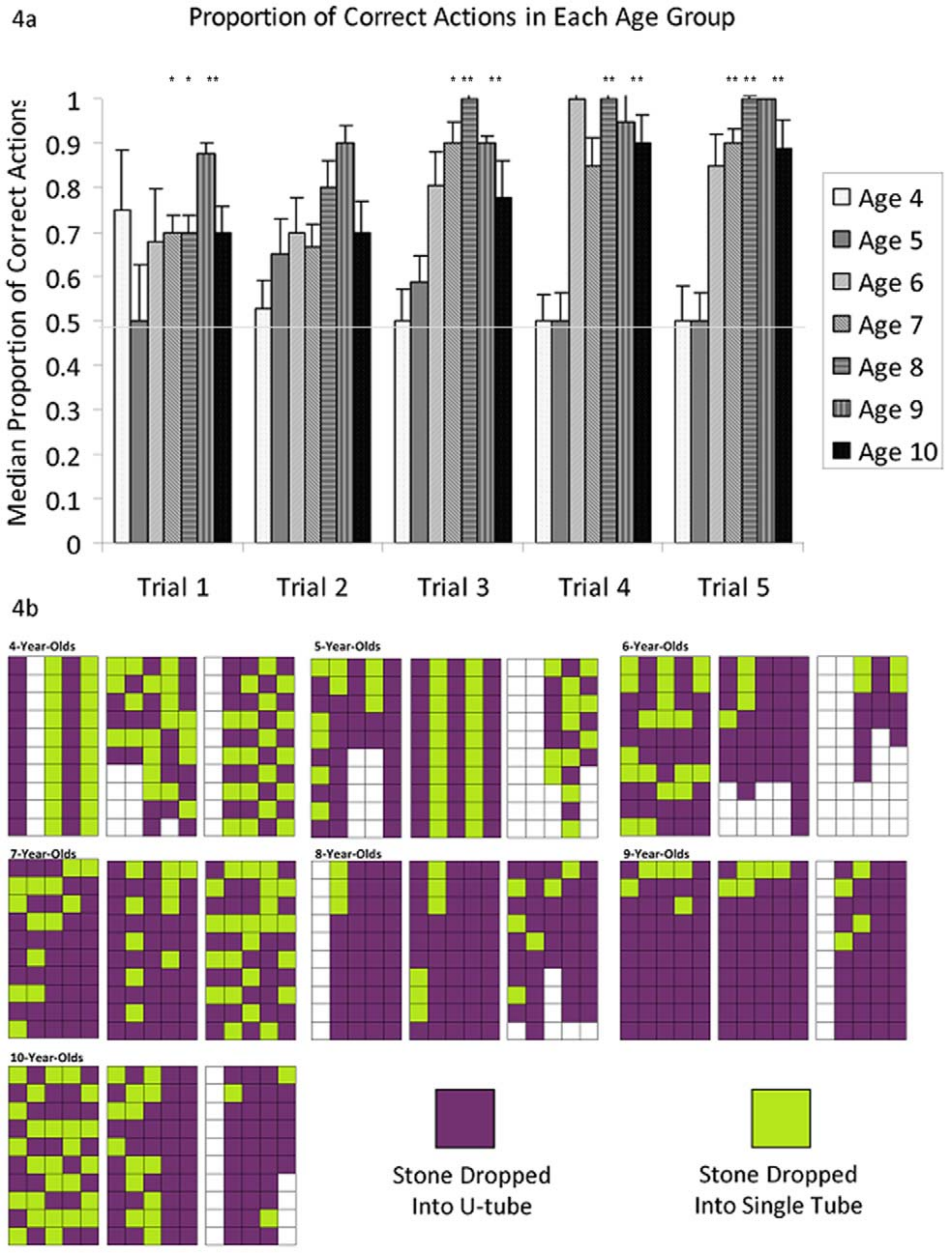
Performance of Children on Task 3. 4a. shows the median performance of children of different age groups across the five trials of Task 3. Error bars represent 95% confidence intervals. 4b shows the individual stone insertions of 3 children chosen at random from each age cohort. Each column represents the order in which items were inserted within a single trial. Stars represent trials in which that age group performed above chance according to one-sample wilcoxen (5-year-olds: 2^nd^: W = 11, n = 10, p>0.5; 3^rd^: W = 27, n = 10, p>0.1; 4^th^: W = 7, n = 9, p>0.05; 5^th^: W = 14, n = 10, p>0.1; 7-year-olds: 1^st^: W = 45, n = 9, p<0.05; 2^nd^: W = 37, n = 10, p>0.05; 3^rd^: W = 56, n = 11, p<0.02; 4^th^: W = 38, n = 10, p>0.1; 5^th^: W = 91, n = 13, p<0.002; 8-year-olds: 1^st^: W = 21, n = 6, p = 0.05; 2^nd^: W = 43, n = 11, p>0.05; 3^rd^: W = 55, n = 10, p<0.01; 4^th^: W = 62, n = 11, p<0.01; 5^th^: W = 55, n = 10, p<0.01: 9/10-year-olds: 1^st^: W = 36, n = 9, p<0.05; 2^nd^: W = 45, n = 9, p<0.005; 3^rd^: W = 76, n = 12, p<0.005; 4^th^: W = 60, n = 12, p<0.02; 5^th^: W = 78, n = 12, p<0.005)

Performance on this task was similar to Tasks 1 and 2. Again, performance improved with age and trials (Fig 4). The proportion of stones inserted into the correct tube was compared to a chance level of 0.5 for each age group for each trial using one-sample Wilcoxen signed ranks tests (exact statistics reported in figure 4). Due to sample size constraints, performance against chance was not calculated for the 6-year-olds and the 9/10 year-old groups were combined. Chance was also not calculated for the 4-year-olds and the first trial of the 5-year-olds because the majority of scores were exactly 0.5. Wilcoxon analyses discount “matching” pairs from the dataset and thus the sample size was reduced to under 5. The 5-year-olds did not perform above chance in any trial, the 7-year-olds performed above chance in all trials except the 2^nd^ and 4^th^, the 8-year olds performed above chance in all except the 2nd trial and the 9-/10-year-olds performed above chance in all trials.

Age correlated with performance only in the 4th and 5th trials (Kendall's Tau: Trial 4: R(63) = 0.291, p<0.005; trial 5: R(62) = 0.266, p<0.01). There was an effect of age on performance in only the 4th trial (p<0.05). Post-hoc pairwise comparisons revealed a significant difference between 4- and 8-year-olds (Mann-Whitney U test with Šidák correction: p<0.001) and between 4- and 9-year-olds (Mann-Whitney U test with Šidák correction: p<0.001) on the 4th trial only.

**Figure 5:**
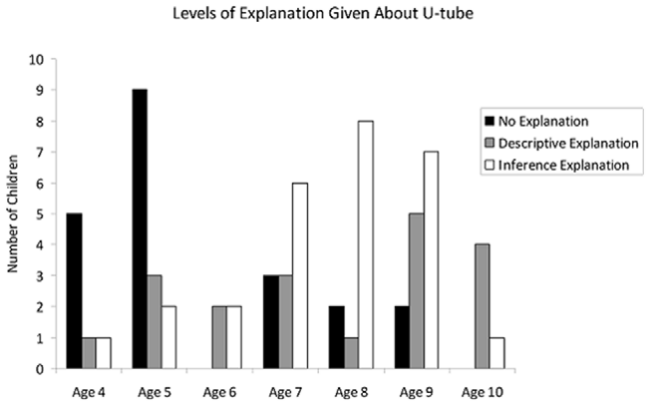
Number of children in each age group that offered each level of explanation.

In answer to the question “how do you think this works?”, no children offered “Mechanistic Explanations”, 25 children offered “Inference Explanations”, 16 children offered a “Descriptive Explanation” and 19 children offered no explanation at all (see Table 2 for examples). Children who offered descriptive explanations were significantly older than children who offered no explanation (independent samples t-tests t(33) = 3.528, p<0.001; means 7.89 and 5.83 respectively; see Fig. 5). However, there was no difference in the age of children who offered an inference explanation and those who offered a descriptive explanation (independent samples t-tests t(37) = 0.21, p>0.8; means 8.02 and 7.89 respectively). Obviously verbal and general cognitive development will account for much of the difference between individuals in their reports; children may be able to *understand* a concept, but not verbally able to report it. Nonetheless, such verbal reports are informative as to children's thought processes.

**Table 2:** Examples of answers given to the question “How do you think it works?”

Type of Explanation	Age of Child	Explanation Offered
**No Explanation **Children do *not* describe any association between action and outcome.	4-Years - 4-Years	“Green and Purple” - “Dunno”
**Description Explanation **Children *describe* the relationship, but offer no explanation.	5-Years - 7-Years - 7-Years	“Green makes water go down. Purple makes water go up.” “One tube makes it go higher, the other doesn't, dunno why.” “This one makes the middle rise, this one doesn't do anything.”
**Inference Explanation **Children offer an *explanation* that involves a connection between the tubes.	8-Years - 8-Years	“The purple one has a connecting pipe – pushes it down, makes it rise. The green one has no connecting pipe.” “Purple works, not Green one. There's water underneath – stops the pebbles, makes water rise in the middle tube.”

Answers were coded by two observers, who had an 89% concordance rate. Children who said nothing were not included, children who spoke but did not describe were coded as “no explanation”, children who described some connection between their action and the outcome, but offered no explanation were coded as “description explanation” and those that mentioned connectivity or “pushing” were coded as “inference explanation”.

The proportion of stones dropped into the correct tube (the U-tube) by children who offered different types of explanation was compared to a chance level of 0.5 using one-sample Wilcoxon signed rank tests. Children who offered no explanation did not perform above chance in any trials (1^st^: W = 20, n = 8, p>0.05; 3^rd^: −19, n = 10, p>0.1; 4^th^: W = 6, n = 9, p>0.05; 5^th^: W = −7, n = 10, p>0.5). Children who offered a descriptive explanation performed above chance in all trials (1^st^: W = 28, n = 7, p<0.02; 2^nd^: W = 45, n = 9, p<0.01; 3^rd^: W = 58, n = 12, p<0.05; 4^th^: W = 103, n = 14, p<0.002; 4^th^: W = 74, n = 15, p<0.05). Children who offered an inference explanation performed above chance in all trials (1^st^: W = 78, n = 12, p<0.005; 2^nd^: W = 77, n = 14, p<0.02; 3^rd^: W = 231, n = 21, p<0.001; 4^th^: W = 231, n(/r) = 21, p<0.001; 5^th^: W = 247, n = 22, p<0.0001). There was a significant effect of level of explanation on performance in all 5 trials (independent samples Kruskal-Wallis Test: trial 1; p<0.001, trial 2: p<0.05, trial 3: p<0.001, trial 4: p<0.001, trial 5: p<0.001).

Taken together, the data on performance on the U-tube task suggests that, as in the previous experiments, children from 8 years were able to learn within a single trial which tube they should drop stone into to cause the token to rise, even when the mechanism was hidden (and potentially “counter-intuitive”). Younger children struggled with the task, but 7-year-olds could learn over 5 trials. The children's ability to pass this task appears to depend not on their capacity to infer the presence of a hidden U-tube, but on their ability to notice and describe the causal relationship between a particular action and the approach of the token.

### Analysis across Tasks

Data from all children in all three tasks was entered into a Generalised Estimating Equations model with a binary logistic response. The model included age (in years) and level of explanation as between subjects factors, and trial and task as within subjects factors. The model also included the following interactions: age × trial, age × task, explanation × age, explanation × trial, explanation × task, trial × task. The dependant variable was proportion of correct actions (i.e. marbles/stones dropped into the correct tube, or correct item dropped into the water tube) out of total actions performed (i.e. total number of items dropped).

**Figure 6:**
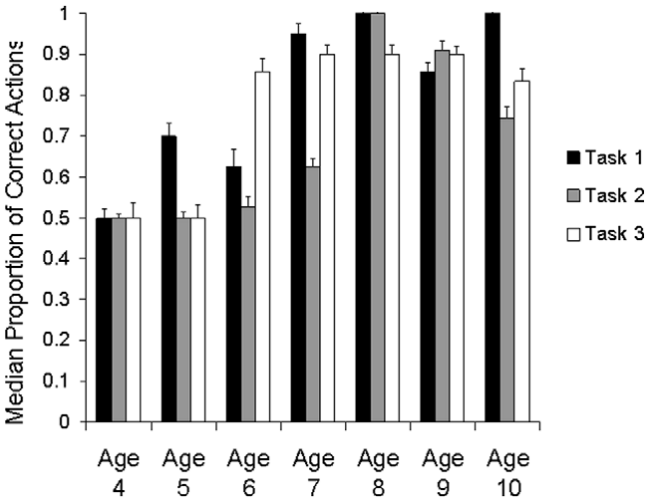
Pattern of performance of children of each age group on each task. Error Bars represent 95% confidence intervals.

The model found a main effect of Age (in years) (χ^2^(6) = 120.752, p<0.001), task (χ^2^(2) = 14.152, p<0.001), trial (χ^2^(4) = 25.269, p<0.001) and level of explanation (χ^2^(2) = 13.061, p<0.001). Children of different ages were also shown to improve over trials at different rates (age × trial interaction: χ^2^(24) = 93.214, p<0.001) and offer different levels of explanation (level of explanation × age interaction (χ^2^(8) = 48.136, p<0.001). Children with different levels of explanation were shown to improve over trials at different rates (explanation × trial interaction: χ^2^(8) = 28.669, p<0.001) and perform differently on different tasks (level of explanation × task interaction: χ^2^(4) = 15.0, p<0.01). However, children of different ages did not perform differently on different tasks (no age × task interaction (χ^2^(12) = 13.932, p>0.3, see Fig. 6) and children did not to improve over trials at different rates in different tasks (no trial × task interaction: χ^2^(8) = 8.644, p>0.3). Given that there was an interaction between age and level of explanation, and between both these factors and trial, it would have been ideal to investigate the interaction between these three factors. However, there were not sufficient degrees of freedom to split the data any further.

These effects were further explored with a series of post-hoc investigations. Performance did not differ between the tasks. There was no significant difference between performance on any of the tasks (Wilcoxon signed ranks tests: task 1–2: p>0.2, task 1–3: p<0.9, task 2–3: p>0.5). This remained the case even if only data from children who did not infer the presence of the U-tube in Task 3 were included (Friedman ANOVA: p>0.3). Looking at the tasks together, performance improved significantly over trials: performance on trial 1 differed from performance on trials 4 and 5 (Mann Whitney U tests: p<0.001 and p<0.005 respectively) and performance on trial 2 also differed from performance on trial 5 (Mann Whitney U test: p<0.005).

Children's overall performance improved with age, but there was not an increase in learning over trials (as measured by the difference in proportion of correct actions between trials 1 and 5) with age. There was a strong positive correlation between age and the proportion of correct actions (Kendell's tau: R(41) = 0.750, p<0.001) but no correlation between age and learning (Kendall's tau: R = −0.068, p>0.4). These results suggest that while the main effect of age was due to a relatively linear improvement in performance, this was not the case with the learning effect. Given the poor performance of the youngest children and the almost perfect performance of the oldest children, this might suggest that the younger children were less able to learn across the five trials, while the older children did not need to because their performance was good from the first trial.

The children who offered “Inference Explanations” scored better overall than those that offered “No Explanations” (Mann Whitney U test with Šidák correction: p<0.001). No other comparisons regarding the levels of explanation were significant. Children who offered “Descriptive Explanations” improved over trials to a different degree to children who offered no explanation (Mann Whitney U with Šidák correction: p<0.017) Children's level of explanation had an impact on scores only in tasks 2 and 3 (Mann Whitney U test with Šidák correction; p<0.001 and p<0.001 respectively). This pattern of results suggests that while performance on Task 1 did not depend on the ability to infer unobservable events, or even describe causal relationships, performance on Tasks 2 and 3 did relate to these factors.

### Comparison with Piagetian Measure

There was no significant difference between children that did or did not pass the conservation test in any of the tasks, although there was a trend suggesting conservers performed better than non-conservers on the U-tube task (task 1: t(63) = 0.709, p = 0.481; task 2: t(63) = 0.018, p = 0.985; task 3: t(52) = −1.802, p = 0.077).

## Discussion

Our data indicate that children have, by the age of 8 years, developed a sophisticated understanding of the relationship between sinking objects and the resulting change in the level of liquid. These children performed above chance from the 1st trial in all tasks. Children between 4 and 7 years were able to learn within 5 trials to drop marbles into water, rather than sawdust, to raise its level, and children between 5 and 7 years were able to learn within 5 trials to drop sinking, rather than floating, items into water to raise its level. There was a suggestion that the younger children (ages 4–5-years) learned more slowly than older children (7-years), while children of 8-years and older were able to learn within the first trial. This may suggest that instrumental conditioning ability improves gradually across these age groups, and is, by 8-years, an extremely fast and effective learning mechanism. There was no significant difference between performance on these tasks and performance on a task designed to present “confusing” physical cues. This was the same across all age groups (as indicated by the lack of age × task interaction) and was not the result of children inferring the presence of the U-tube. This is counter to the hypotheses outlined in the introduction, which predicted that children of between 7 and 9 years would have a rudimentary understanding of the mechanisms underlying raising the water level, and therefore be less able to perform on tasks in which dropping a stone into one body of water apparently causes the level of an adjacent body of water to rise (Task 3). It should be noted at this point that the tasks were conducted in a fixed order by all subjects to maintain comparability with the corvids. The extent to which the experience of tasks 1 and 2 helped, hindered, or was necessary for performance in task 3 was thus not investigated, although this would make an interesting topic of future study.

The performance of children on the first two tasks is comparable to that of the three corvid species studied. Rooks, Eurasian Jays and New Caledonian Crows were all able to learn to drop stones into water rather than sawdust within about 5 trials, a performance that equates roughly with the 4–7-year-old children tested here. Eurasian Jays and New Caledonian Crows were able to learn to drop sinking items into water rather than floating items within 5 trials, a performance that equates roughly with the 5–7 year-old children.

Such comparisons are useful only to the extent to which we are able to investigate the possible presence of some common mechanism by which the children and corvids may be solving such tasks. As such, an interesting difference between the performance of the corvids and the children emerges in Task 3 (The U-tube Task). The Eurasian Jays tested performed substantially worse on Task 3 than on Tasks 1 and 2. This was taken by Cheke and colleagues [2] to suggest that the birds had a rudimentary concept of the causal mechanism underlying the relationship between their stone dropping and the movement of the reward, and the causal relationship in the U-tube task violated the assumptions of what was possible according to this mechanism. The children's performance was equivalent on this task to the other tasks, even in those individuals that did not infer the presence of the U-tube. The children who were successful on the U-tube task were those that were able to notice and describe the causal relationships between putting a stone in a particular place and the approach of the food. These children could be said to be learning using a model of instrumental learning suggested by Cheke and colleagues in the Eurasian Jay paper [2]: Model D: Do the action that causes the movement of the reward. In contrast, the Jays' performance was more in line with Model E: Do the action that causes the movement of the reward, where the choice of action is affected by, but not reliant on, some concept of mechanism.

The fact that children were not impaired on the U-tube task relative to the other tasks may indicate that the they did not interpret the event as “impossible” because they did not understand that insertion of an item into one body of water cannot raise the level of another body of water. More likely, the children simply ignored the “impossible” causal cues. Indeed, it has been found that children as old as 11-year-old prioritise contingency and contiguity as evidence of causality above information about mechanism and may ignore information about mechanism altogether if this conflicts with apparent contingency information [13], [21], [22]. When applied to the current results this finding might suggest that, due to the robust covariation, children's willingness to believe their actions to be causal was not impacted by the presence of cues indicating that the token was not in the same body of water as the stones. On the other hand, it may be that children with no comprehension of the mechanism did not explicitly attribute causation, but simply allowed covaration information to guide their actions.

That a bias to prioritise co-variation above mechanism information exists in children is extremely interesting and worthy of further investigation. It may be that it is a useful developmental stage which exists to allow children to learn about causation unfettered by ideas of what is and is not “possible”. On the other hand, such a bias could conceivably come about as a product of extensive technological enculturation: children have considerable experience of devices with hidden mechanisms that make apparently impossible events happen (e.g. computers, light switches) and for which children are not encouraged to investigate mechanism (indeed, many adults do not understand anything about the mechanism) but simply learn to use relying on covariation between action and outcome. It may be that, for children growing up in heavily mechanised/electrically powered societies, understanding causal mechanisms has become somewhat separated from the development of causal judgements. To separate these two points, it might be interesting to study children and adults from both mechanised and non-mechanised cultures to discover in which populations this bias exists.

With regard to the present study, it should be noted that children needed considerable probing on the U-tube task to persuade them to do anything other than attempt to insert the stones into the tube containing the token and in some cases might never have done so without being explicitly told to “try the other tubes”. This was done because we were interested in their learning, not their ability to disengage from a target. However, it may be that this encouragement caused the children to think that “something would happen to the token” if they dropped a stone into one of the other tubes, and therefore to look between them to check. There is evidence that young children will ignore the evidence of their own eyes if given conflicting testimony by an adult [23]. This idea that dropping a stone into an apparently unconnected body of water might alter the position of the target might have been exactly what was missing in the birds – they may simply not have looked in the correct place to see the result of their actions. Furthermore the school setting and that the task was presented by an adult may have led the children to trust that there *must be* a solution, and to accept that they may not understand it; this is after all a common occurrence in the classroom.

In general the comparison between the performance of the corvids and children should be undertaken with extreme caution. One cannot easily compare the results from a few individuals (as with the birds) to a large cohort (as with the children). Furthermore time constraints meant that the children had only 5 trials at each task and undertook all three tasks in the space of an hour, while the birds had 15 trials and undertook the tasks over several days. It may be that the differences between the tasks similar to the birds might emerge in children if given more trials, or a longer break between tasks. The size and morphological differences between the birds and children should also be noted; having two hands meant that the children had access to different advantageous (e.g. pushing down on floating items to force them under the water) and disadvantageous (inserting two stones simultaneously into both tubes in Task 3 such that it was impossible to know which raised the token) strategies, which will have had a considerable impact on their learning. Furthermore, a bird's beak is very much closer to its eyes than a child's hands are to theirs. This difference has a substantial impact on what cues these groups can observe while conducting the task. Finally, while the corvids were working for primary reinforcers, the children were working for tokens which could be swapped, which may have led to differences in motivation. Nonetheless, despite these caveats, we believe that there is much to be learned from performing comparable tests in different species.

It is interesting to note that children only started reliably performing above chance on any of the tasks by the age of 8. This is in line with recent findings that children only reliably perform “intuitive” problem solving using tools at around 7–8 years [17], [18], and with the age at which children tend to pass the Piagetian volume conservation task [15]. However there are two surprising elements. First, that this task, which does not require such “insightful” re-evaluation of a situation but instead uses a pre-trained motor action and gives clear co-variation cues, should be passed so late. Specifically, much later than the False Belief Task (which is passed at around 4-years), which involves reasoning about complex unobservable causal mechanisms (i.e. beliefs). This apparently paradoxical developmental asynchrony may be the result of the differential attention and weight given to social and physical information: consider the finding of Whiten and colleagues [24], [25] that young children (and indeed adults [26]) will imitate the actions of a demonstrator even when they are obviously causally irrelevant. Children are constantly encouraged to consider human behaviour in terms of the underlying mechanism (e.g. emotions, beliefs) but are rarely encouraged to do so in the same way with machines. It is furthermore surprising that there was little to no relationship between performance on these experiments and on the conservation task. However, this could be interpreted as further evidence that children solved the tasks using covariation cues alone, rather than drawing on their knowledge of the nature of the underlying mechanism.

In summary, children between 4 and 10 years of age were tested on a tool-use task developed with corvids [1], [2], [3]. Although children from 4-years were able to learn to drop marbles into water rather than sawdust to raise the level of a token over the course of 5 trials, this task was only solved reliably by children over 8 years of age. Children from 5-years were able to learn to drop marbles, rather than cork balls, into water to raise the level of a token over the course of 5 trials. Again this task was only solved reliably by children over 8-years of age. Finally, children did not perform relatively worse on a task in which dropping a stone into one tube of water apparently caused a rise in level of the token floating in an adjacent tube of water. This result is in contrast to the corvid findings and may support previous research that children will ignore information regarding mechanism in favour of using co-variation as evidence of causation, or to guide their actions when causation is uncertain.

## Supporting Information

Supporting Information S1 Token Retrieval. The success in retrieving the token was recorded. The purpose of the study was to report on the learning process and on whether children learned to perform and repeat the effective action over the ineffective action. The number of effective actions performed was thus the variable studied. The success at retrieving the token is a far messier measure since it depends on so many other factors (children's token-extraction technique, children's motivation). Given the number of analyses already in the study, it was decided not to conduct in-depth analyses into token-retrieval.
(DOC)
