# Supplementary material for: How Do Children Solve Aesop's Fable?
Source: PLoS One. 2012 Jul 25;7(7):e40574. doi: 10.1371/journal.pone.0040574 (PMC3405099; doi:10.1371/journal.pone.0040574)
Supplement: Supporting Information S1 — Token Retrieval. The success in retrieving the token was recorded. The purpose of the study was to report on the learning process and on whether children learned to perform and repeat the effective action over the ineffective action. The number of effective actions performed was thus the variable studied. The success at retrieving the token is a far messier measure since it depends on so many other factors (children's token-extraction technique, children's motivation). Given the number of analyses already in the study, it was decided not to conduct in-depth analyses into token-retrieval. (DOC) [file pone.0040574.s001.doc]

S1

Task 1

20 children (3 4-year-olds, 2 5-year-old, 4 7-year-olds, 5 8-year-olds, 3 9-year-olds and 3 10-year-olds) retrieved the token on the first trial, 18 children (2 4-year-olds, 3 5-year-olds, 2 7-year-olds, 7 8-year-olds, 2 9-year-olds and 2 10-year-olds) retrieved the token on trial 2, 33 children (4 4-year-olds, 7 5-year-olds, 1 6-year-old, 7 7-year-olds, 6 8-year-olds, 5 9-year-olds and 3 10-year-olds) retrieved the token on trial 3, 33 children (6 4-year-olds, 9 5-year-olds, 2 6-year-olds, 4 7-year-olds, 6 8-year-olds, 4 9-year-olds and 2 10-year-olds) retrieved the token on trial 4 and 33 children (5 4-year-olds, 8 5-year-olds, 2 6-year-olds, 7 7-year-olds, 6 8-year-olds, 3 9-year-olds and 2 10-year-olds) retrieved the token on trial 5.

Task 2

Only one child (aged 10) retrieved the token on trial 1, 19 children (1 5-year-old, 1-6-year-old, 3 7-year-olds, 7 8-year-olds, 5 9-year-olds and 2 10-year-olds) retrieved the token on trial 2, 17 children (1 4-year-old, 2 5-year-olds, 2 6-year-olds, 2 7-year-olds, 7 8-year-olds and 3 9-year-olds) retrieved the token on trial 3, 27 children (1 4-year-old, 2 5-year-olds, 2 6-year-olds, 7 7-year-olds, 4 8-year-olds and 6 9-year-olds and 2 10-year-olds) retrieved the token on trial 4, 27 children children (1 4-year-old, 2 5-year-olds, 2 6-year-olds, 8 7-year-olds, 8 8-year-olds, 5 9-year-olds and 1 10-year-old).

Task 3

24 children (2 4-year-olds, 1 5-year-old, 10 7-year-olds, 5 8-year-olds, 4 9-year-olds and 2 10-year-olds) retrieved the token on the first trial, 38 children (1 4-year-olds, 6 5-year-olds, 1 6-year-old, 12 7-year-olds, 10 8-year-olds, 6 9-year-olds and 2 10-year-olds) retrieved the token on trial 2, 43 children (1 4-year-old, 7 5-year-olds, 2 6-year-olds, 11 7-year-olds, 10 8-year-olds, 8 9-year-olds and 4 10-year-olds) retrieved the token on trial 3, 45 children (2 4-year-olds, 8 5-year-olds, 2 6-year-olds, 12 7-year-olds, 10 8-year-olds, 7 9-year-olds and 4 10-year-olds) retrieved the token on trial 4 and 44 children (2 4-year-olds, 6 5-year-olds, 2 6-year-olds, 14 7-year-olds, 10 8-year-olds, 7 9-year-olds and 3 10-year-olds) retrieved the token on trial 5.
